# P18 A case report on Atypical Polymyalgia Rheumatica in Young Adult

**DOI:** 10.1093/rap/rkac067.018

**Published:** 2022-09-28

**Authors:** Asif Munir, Rachel Hutchinson

**Affiliations:** North Cumbria Integrated Care NHS Foundation Trust, Carlisle, United Kingdom; North Cumbria Integrated Care NHS Foundation Trust, Carlisle, United Kingdom

## Abstract

**Introduction/Background:**

Polymyalgia Rheumatica is a common inflammatory condition after the age of 50 years. It typically presents with shoulder, neck and pelvic pain and stiffness associated with raised inflammatory markers. We report a case of Polymyalgia Rheumatica in a young male of 40 years in which clinical presentation was not typical.

**Description/Method:**

A 40-year-old male manager in a retail shop was reviewed in Rheumatology Outpatient clinic with a few months history of back and pelvic pain with progressively worsening early morning stiffness for upto 40 minutes associated with marked fatigue ability. There was also pain in the right shoulder following a trauma causing rotator cuff tear shown on MRI done a year ago. His CRP was 19 and ESR 52. Initial assessment was inflammatory Axial Spondyloarthropathy.

MRI of spine and SI joints as discussed in radiology MDT didn’t show inflammatory changes in the spine or SI joints. It showed bilateral Osteonecrosis of Femoral heads and ill-defined oedema in left acetabulum. A repeat MRI and CT of pelvis showed sclerotic lesion in left Acetabulum. The patient was discussed in bone and soft tissue tumour MDT with conclusion of non-malignant lesion without requiring biopsy and seen by orthopaedics.

The pain progressed to involve left shoulder as well despite regular use of NSAIDs. The stiffness deteriorated and became generalised for up to 2 hours. The fatigability also got worse with some interference in his Job. CRP readings were 19, 19, 25 and 20 whereas ESR was 52, 48, 32 and 40 with an interval of a month each.

Multiple other investigations including WCC, Hb, U& Es, LFTs, TSH, CK, bone profile, RF, anti CCP, ANA, ENA, ANCA, Serum protein electrophoresis, Myeloma screen, urine analysis, XR chest and ECG were normal.

He went on to have PET CT that showed increased uptake suggestive of arthritis in both shoulders, sternoclavicular joints, 1st Costochondral joint, left hip and posterior spinal ligament. The findings were discussed in Radiology MDT again with a conclusion of diagnosis of Polymyalgia Rheumatica based on the distribution of joints involved.

**Discussion/Results:**

The patient was started on Prednisolone at a dose of 20mg daily. His symptoms improved within 3 to 4 days of start of Prednisolone. Inflammatory markers also improved were checked after 4 weeks with CRP 4 and ESR 18 and they remained normal in two subsequent readings after two and three months. He is currently on tapering dose of Prednisolone without recurrence of symptoms as well as rise in inflammatory markers.

Pattern of joint involvement on PET CT and dramatic response to steroids confirmed the diagnosis of PMR.

**Key learning points/Conclusion:**

Although PMR is most commonly seen in adults over 50 years but it can present in young adults like in this patient.

Because of young age and atypical presentation mutiple investigations including PET CT was required to reach to the diagnosis of PMR that otherwise is made based on clinical presentation associated with raised raised inflammatory markers.

Osteonecrosis of Femoral heads in the absence of any other positive investigation was thought most likely secondary to Inflammation that again is not very common presentation of PMR.

A long term follow up is required for this patient to monitor the response of reducing and then stopping of steroids.

